# GENE-ENVIRONMENT INTERPLAY BETWEEN SMOKING BEHAVIOR AND COGNITION AMONG OLDER ADULTS

**DOI:** 10.1093/geroni/igz038.1255

**Published:** 2019-11-08

**Authors:** Shandell Pahlen, William Kremen, Chandra A Reynolds

**Affiliations:** 1 University of California, Riverside, Riverside, California, United States; 2 University of California, San Diego, San Diego, California, United States

## Abstract

Associations between smoking behavior and lower cognitive functioning have been observed but there is a paucity of evidence examining the etiological impact of smoking on cognition. The current study explored the moderation of genetic and environmental contributions to cognition across mid and late-adulthood by smoking behaviors in 8 twin studies from the international IGEMS consortium (N=11,764; Mage=63.1 years). Mixed effects regression models between smoking behavior and cognition found the strongest negative effects for smoking on Symbol Digit (Bpackyears=-1.42, p<.0001) and Block Design (Bpackyears=-1.79, p=.0008), while controlling for dependency between twin siblings, age, sex, and country. Although the negative effects tended to be more pronounced for males, we did not find significant sex moderation. Univariate biometric models considered smoking behavior (status and pack years) and age as moderators of genetic and environmental components contributing to cognitive performance. Results for both Symbol Digit and Block Design suggest that smoking (current and past) is associated with lower genetic, and higher environmental influences on cognition compared to non-smoking. For Block Design, but not for Symbol Digit, pack years moderated shared environmental contributions, with the highest contributions found for current smokers compared to former. Overall, results illustrate an increasing saliency of smoking related environmental influences for processing speed and spatial reasoning tasks. Cognitive tasks with speed components may be sensitive to age-related declines, and speed may also represent a factor vulnerable to smoking exposure, potentially implicating important health and neurobiological pathways. Supported by NIH Grant Nos. R56 AG037985, R01 AG060470.

